# Targeted LC-MS/MS platform for the comprehensive determination of peptides in the kallikrein-kinin system

**DOI:** 10.1007/s00216-021-03231-9

**Published:** 2021-03-10

**Authors:** Tanja Gangnus, Bjoern B. Burckhardt

**Affiliations:** Institute of Clinical Pharmacy and Pharmacotherapy, Heinrich Heine University, 40225 Dusseldorf, Germany

**Keywords:** Bradykinin, Kallikrein-kinin system, LC-MS/MS, Plasma, Angioedema, COVID-19

## Abstract

The kallikrein-kinin system (KKS) is involved in many physiological and pathophysiological processes and is assumed to be connected to the development of clinical symptoms of angioedema or COVID-19, among other diseases. However, despite its diverse role in the regulation of physiological and pathophysiological functions, knowledge about the KKS in vivo remains limited. The short half-lives of kinins, their low abundance and structural similarities and the artificial generation of the kinin bradykinin greatly hinder reliable and accurate determination of kinin levels in plasma. To address these issues, a sensitive LC-MS/MS platform for the comprehensive and simultaneous determination of the four active kinins bradykinin, kallidin, des-Arg(9)-bradykinin and des-Arg(10)-kallidin and their major metabolites bradykinin 2-9, bradykinin 1-7 and bradykinin 1-5 was developed. This platform was validated according to the bioanalytical guideline of the US Food and Drug Administration regarding linearity, accuracy, precision, sensitivity, carry-over, recovery, parallelism, matrix effects and stability in plasma of healthy volunteers. The validated platform encompassed a broad calibration curve range from 2.0–15.3 pg/mL (depending on the kinin) up to 1000 pg/mL, covering the expected concentrations in disease states. No source-dependent matrix effects were identified, and suitable stability of the analytes in plasma was observed. The applicability of the developed platform was proven by the determination of endogenous levels in healthy volunteers, whose plasma kinin levels were successfully detected in the low pg/mL range. The established platform facilitates the investigation of kinin-mediated diseases (e.g. angioedema, COVID-19) and enables the assessment of the impact of altered enzyme activities on the formation or degradation of kinins.

**Graphical abstract:**

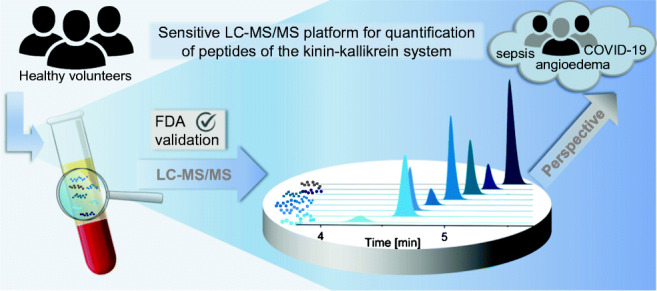

## Introduction

**Fig. 1:**
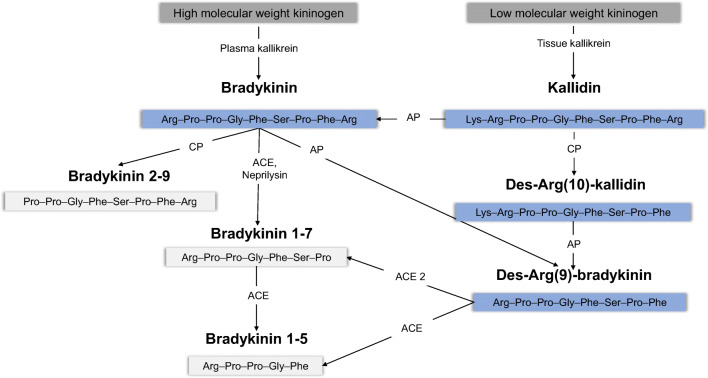
Overview of the kallikrein-kinin system. Active kinins are marked in blue and inactive kinins in light grey. The amino acid sequence of each peptide is displayed in the three-letter amino acid code. ACE, angiotensin-converting enzyme; AP, aminopeptidase; CP, carboxypeptidase

The kallikrein-kinin system (KKS) is involved in many physiological and pathophysiological processes, including regulation of blood pressure, cardiac function, renal function, inflammation, pain and cough [1, 2]. Clinical conditions to which kinins are connected comprise angioedema and angiotensin-converting enzyme (ACE) inhibitor–induced cough, in which elevated levels of the kinin bradykinin are postulated [3, 4]. Furthermore, the so-called bradykinin storm has been implicated in the ongoing COVID-19 pandemic, as it has been proposed to lead to severe COVID-19 pathologies [5]. The active kinins bradykinin and kallidin are produced by proteolytic cleavage from kininogen through the action of plasma and tissue kallikrein, respectively (Fig. 1) [6]. Moreover, bradykinin can be generated by aminopeptidase P–mediated cleavage of kallidin. Bradykinin and kallidin, along with their des-Arg metabolites, des-Arg(9)-bradykinin and des-Arg(10)-kallidin, contribute to the biological activity of kinins in humans. These active kinins are degraded by distinct enzymes (e.g. ACE, ACE 2, carboxypeptidase N or aminopeptidase P) into inactive metabolites (e.g. bradykinin 1-7, bradykinin 1-5) in plasma (Fig. 1) [7, 8]. During COVID-19, a dysregulated KKS is hypothesised to be caused by reduced des-Arg(9)-bradykinin degradation through virus-associated ACE 2 inhibition on the one hand and increased bradykinin production via alternative cleavage of bradykinin precursors or induction of the bradykinin-forming enzyme kallikrein on the other hand [5, 9–11]. Confirmation of these postulated alterations requires a comprehensive quantitative determination of the KKS peptide levels.

However, despite its important physiological and pathophysiological roles, the current in vivo knowledge about the KKS remains limited, as the reliable and accurate determination of peptides of the KKS in plasma is a major hurdle. Artefactual changes of endogenous levels owing to rapid enzymatic degradation or artificial generation of bradykinin through contact with surfaces need to be prevented by an adequate protease inhibitor [12–14]. Furthermore, their low endogenous concentrations of few pg/mL and the structural similarity of kinins with their precursors and metabolites call for sensitive and specific assays [2]. These confounding factors have contributed to diverging reported ranges of kinin levels. Immunometric detection–based assays claim low detection limits of 0.5–1.4 pg/mL for bradykinin [15–17]. Using these assays, reported endogenous levels ranged between 1.7 and 15.8 pg/mL for bradykinin in healthy volunteers. However, the disadvantage of immunoassays is that they commonly suffer from cross-reactivities with related kinins. Liquid chromatography coupled to mass spectrometry (LC-MS/MS) can overcome these specificity issues. Approaches towards quantification of several kinins simultaneously by nano LC-MS/MS indicated the high potential of this technique [18]. Nevertheless, reported levels of bradykinin applying available LC-MS/MS assays still vary from 200 pg/mL [19, 20] up to 160 ng/mL [21] in healthy volunteers. Consequently, other studies have focused on the determination of only the more stable metabolite bradykinin 1-5, whose levels are unaffected by the artificial generation of bradykinin if the degradation of bradykinin is sufficiently inhibited [22, 23]. However, this approach only delivers limited information as the alterations in the KKS cascade are dominated by different concomitant metabolic pathways leading to the formation of bradykinin 1-5. Moreover, LC-MS/MS offers a tool for the determination of multiple peptides simultaneously, representing an advantage over immunoassays. Measurement of all active kinin peptides and their major metabolites in one method would allow for the collection of more information and a more comprehensive picture of the status of the KKS than the measurement of single kinins (e.g. bradykinin 1-5). For this purpose, a platform technology is required to facilitate the thorough monitoring of the KKS peptides, as well as their comprehensive investigation in patient groups in which the KKS has been implicated.

Thus, this study aimed to develop, validate and establish a targeted LC-MS/MS platform and demonstrate its applicability for the determination of plasma levels in healthy volunteers for the following kinin peptides: bradykinin, kallidin, des-Arg(9)-bradykinin, des-Arg(10)-kallidin, bradykinin 1-7, bradykinin 1-5 and bradykinin 2-9. The comprehensive investigation of the entire kinin peptide cascade was aspired at facilitating a better understanding of the impact of altered enzyme activities on the presence of active kinins within the KKS in disease.

## Materials and methods

### Preparation of stock and working solutions

Kallidin trifluoroacetic acid (TFA) salt (96.9%, HPLC; Tocris, Bristol, UK), bradykinin acetate (99.0%, HPLC; Sigma-Aldrich, St. Louis, MO, USA) and their metabolites des-Arg(9)-bradykinin acetate (98.7%, HPLC; Santa Cruz Biotechnology, Dallas, TX, USA), bradykinin 1-7 TFA salt (≥95.0%, HPLC; GenScript, Piscataway Township, NJ, USA), bradykinin 1-5 TFA salt (≥95.0%, HPLC; GenScript), bradykinin 2-9 TFA salt (≥95.0%, HPLC; GenScript) and des-Arg(10)-kallidin TFA salt (95.9%, HPLC; Tocris) were dissolved and diluted separately in 0.3% TFA in 25/75 acetonitrile/water (v/v/v) prior to the preparation of a combined working solution containing 400 ng/mL of each peptide (free base). All peptide concentrations given within this study were corrected for salt content and peptide purity referring to the conducted amino acid analysis. [Phe^8^Ψ(CH-NH)-Arg^9^]-bradykinin TFA salt (97.5%, HPLC; Tocris), the internal standard, was dissolved in 0.1% formic acid in water (v/v) and subsequently diluted to achieve a working solution of 500 ng/mL in 0.3% TFA in 25/75 acetonitrile/water (v/v/v). All peptide solutions were prepared using low protein binding tubes (Sarstedt, Nümbrecht, Germany).

### Human blood samples

Blood samples were donated by healthy volunteers and sampled in S-Monovettes® (Sarstedt, Nümbrecht, Germany) containing 1.6 mg/mL ethylenediaminetetraacetic acid (EDTA) or 0.106 mol/L trisodium citrate. All participants gave written informed consent prior to their enrolment. The study was conducted in accordance with the Declaration of Helsinki and approved by the ethics committee of the medical faculty at the Heinrich Heine University (study number: 6112).

### Sample preparation

In this study, human blank plasma was generated by sampling blood into trisodium citrate S-Monovettes® spiked with hexadimethrine bromide and nafamostat mesylate to prevent the artificial generation of bradykinin. After centrifugation at 2000×*g* for 10 min at room temperature, the plasma was left at room temperature for 4 h to enable degradation of short-lived kinins. The blank plasma generated was stored at −20 °C until use.

Before preparation of quality control (QC) or calibration curve samples, a protease inhibitor was added to blank plasma samples, following a protocol derived from Nussberger et al. [24]. Solid-phase extraction (SPE) was performed using 96-well Oasis weak cation exchange (WCX) μ-elution plates (Waters, Milford, MA, USA). The wells were conditioned with 200 μL methanol, followed by 200 μL water. Subsequently, all cartridges were prefilled with 150 μL of 3 ng/mL internal standard in 8% phosphoric acid (v/v) before loading 150 μL of plasma sample. Washing was performed using 300 μL of 25 mM phosphate buffer, followed by 300 μL water and 300 μL 10% methanol in water (v/v). Elution was conducted three times with 50 μL 1% TFA in 75/25 acetonitrile/water (v/v/v). The resulting eluate was evaporated to dryness under a gentle stream of nitrogen at 60 °C while shaking at 300 rpm. The residue was dissolved in 75 μL of 10/10/80 formic acid/methanol/water (v/v/v).

### LC-MS/MS conditions

An Agilent 1200 SL series system (Agilent Technologies, Ratingen, Germany) equipped with a degasser (G1379B), a binary pump SL (G1379B) and a column oven TCC SL (G1316B) was used. For chromatographic separation, a Phenomenex Synergi™ 2.5-μm Hydro-RP 100-Å column (100 × 2.0 mm; Torrance, CA, USA) with an AQ C18 (4.0 × 2.0 mm) security cartridge was applied. The mobile phases consisted of water and methanol (B), both containing 3.2% dimethyl sulfoxide and 0.1% formic acid (v/v). A 7.5 min binary gradient at a flow rate of 0.4 mL/min and a column oven temperature of 60 °C was applied as follows: 0–1.5 min: 5% B, 1.5–2.2 min: 5–20% B, 2.2–2.7 min: 20–27% B, 2.7–3.1 min: 27–35% B, 3.1–6.2 min: 35–95% B, 6.2–6.7 min: 95% B, 6.7–7.5 min: 95–5% B. Thereafter, the column was re-equilibrated for 3 min. The injection volume of 50 μL was applied with a PAL HTC-xt autosampler (CTC Analytics AG, Zwingen, Switzerland), and samples were stored at 18 °C.

**Fig. 2:**
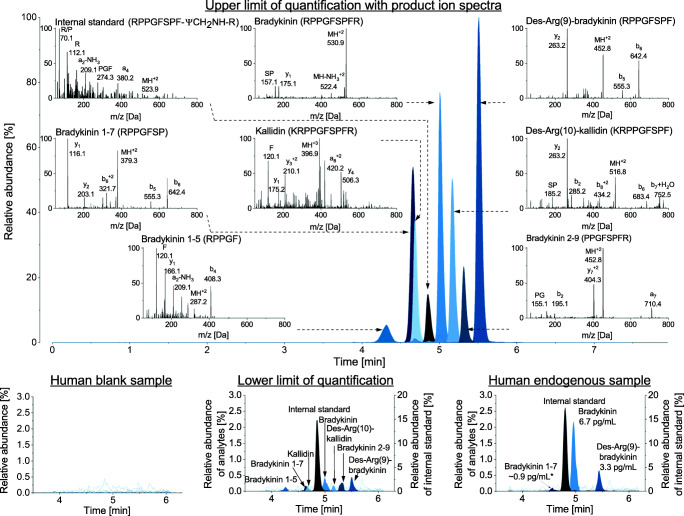
Example chromatograms of the blank, the lower and upper limit of quantification and one endogenous sample of a male volunteer. In the presentation of the upper limit of quantification, the product ion scans of all analytes and the internal standard are additionally displayed with the main ion fragments. The corresponding amino acid code for each peptide is given in the top right of each scan. *extrapolated level below the quantification limit; IS, internal standard

The LC system was coupled to an API 4000 mass spectrometer (AB Sciex, Darmstadt, Germany) equipped with a Turbo V source for detection. The electrospray ionisation source was operated in positive mode with multiple reaction monitoring mode. The curtain gas was maintained at 31 psi, the collision gas at 8 psi, the nebuliser gas at 45 psi and the heater gas at 65 psi. The ion spray voltage was set at 5500 V, while the source temperature was 350 °C. Peptide-specific parameters are displayed in Table 1 and the respective product ion spectra of each kinin are shown in Fig. 2. The most intense transition per peptide was used for quantification to allow for highest achievable sensitivity. Since only one transition was used for the quantification, the specificity was ensured by the relative retention time to the internal standard.

**Table 1:** Peptide-specific transitions and voltage parameters for mass spectrometric detection

Analyte	Transition (m/z)	Dwell time (ms)	Declustering potential (V)	Entrance potential (V)	Collision energy (V)	Collision cell exit potential (V)
Kallidin	396.9➔506.3	65	95	9	23	14
Bradykinin	530.9➔522.4	65	120	10	31	14
Des-Arg(9)-bradykinin	452.8➔263.2	75	85	10	22	15
Des-Arg(10)-kallidin	516.8➔752.5	65	100	10	29	11
Bradykinin 2-9	452.8➔404.3	50	120	9	24	11
Bradykinin 1-7	379.3➔642.4	75	63	10	16	10
Bradykinin 1-5	287.2➔408.3	50	61	8	15	11
[Phe^8^Ψ(CH-NH)-Arg^9^]-bradykinin	523.9➔274.3	75	100	12	48	18

*ms* milliseconds, *m/z* mass-to-charge ratio, *V* volt

Controlling and data acquisition were conducted using Analyst® 1.6.2 software (AB Sciex), and data evaluation was performed using Multiquant™ 3.0.2 (AB Sciex).

### Method development

#### Blank plasma generation

Due to the endogenous presence of kinins in human plasma, it was aimed to develop a surrogate matrix mimicking human biological samples at best. Therefore, distinct serine protease inhibitors were evaluated for their ability to prevent the artificial generation of bradykinin after blood sampling. These inhibitors scarcely affect the mainly metalloprotease-mediated degradation of bradykinin; thus, the protocol takes advantage of the enzymatic degradation of kinins in plasma. Therefore, plasma was drawn into prespiked S-Monovettes® and aliquots of the plasma stored at 21 °C were analysed after 30 min, 1.5 h and 4.5 h. The following inhibitors were investigated and compared to plasma without inhibitor (each *n* = 3): 4-(2-aminoethyl)benzolsulfonylfluoride (AEBSF), hexadimethrine bromide, aprotinin, nafamostat mesylate and leupeptin. Additionally, the final blank generation protocol was evaluated for six human sources, whose generated blanks were analysed in triplicate to ensure the depletion of endogenous peptides across multiple sources.

#### Determination of the calibration curve range

Prior to the validation, the lower and upper limits of the calibration curve range (LLOQ/ULOQ) for each analyte were assessed. To improve the LLOQ, various plasma sample volumes between 100 and 300 μL were concentrated after SPE by reconstitution in only 75 μL of the injection solvent. Quantification limits from 0.78 up to 3000 pg/mL using 13 calibrators were investigated for the predefinition of the calibrators and the QC levels.

### Validation

Kinins are potential biomarkers as their role has been described in diverse health and disease states (e.g. angioedema, sepsis). The context of use is to describe the KKS quantitively in healthy versus diseased populations to record data on the alterations of the peptides and evaluate the potential of the kinins as biomarkers. Following the successful validation, the assay is intended to be applied within clinical studies regarding COVID-19. Validation was carried out according to an in-house validation plan that encompasses the bioanalytical guideline of the US Food and Drug Administration (FDA) [25]. Linearity, accuracy, precision, sensitivity, carry-over, recovery, dilution integrity, parallelism and stability were assessed. Additionally, absolute matrix effects and the coefficient of variation (CV) of the internal standard normalised matrix factor were evaluated according to the bioanalytical guideline of the European Medicines Agency [26].

Linearity of the method was determined by measuring the peak area ratio response (analyte area/internal standard area) for three calibration curves in a range from 2.0 pg/mL (LLOQ) to 1000.0 pg/mL (ULOQ). The calibration curve ranges were analysed for each analyte separately. The deviation of the calculated concentration from the nominal concentration (relative error [RE]) was not allowed to exceed ±15%, with the exception of the LLOQ, for which deviations of ±20% were tolerated. A minimum of 6 calibration curve points and ≥ 75% had to fulfil this criterion, and the RE was recalculated for all remaining calibration curve points if a value had to be excluded.

Accuracy and precision were established using five replicates from each QC level (*n* = 4) in three independent runs on three distinct days. Therefore, a QC high (750.0 pg/mL), a QC mid (125.0 pg/mL) and several QC lows/LLOQs (31.1 pg/mL, 15.6 pg/mL, 7.8 pg/mL, 3.9 pg/mL and 2.0 pg/mL) were analysed. Different QC low/LLOQ levels were used, depending on the calibration curve range. Using one-way analysis of variance, intermediate precision (within-run) and the day-different precision (between-run) were calculated and had to be ≤15% (CV) at the distinct QC levels, with the exception of the LLOQ, for which CVs of ≤20% were permitted. Between-run accuracy was calculated as the mean RE of three runs. Accuracy was confirmed if the mean calculated concentrations from the nominal concentrations deviated by ≤15% (RE) at the QC levels and ≤ 20% at the LLOQ. Additionally, the analyte response had to exceed 5 at the LLOQ. The analyte response was determined by the signal-to-noise ratio calculated by the SignalFinder™ integration algorithm using Multiquant™ 3.0.2.

To check for possible carry-over, six ULOQ and six blank samples were consecutively injected. The response of the analytes in the blank samples was not allowed to exceed 20% of the response at the LLOQ and 5% of the response of the internal standard.

Recovery of the analytes was calculated by comparing the mean area response of plasma samples spiked before SPE to blank plasma samples spiked with the analytes after SPE. The following QC levels were assessed using this method: the QC high (750.0 pg/mL), the QC mid (125.0 pg/mL) and two QC lows (31.1 pg/mL and 7.8 pg/mL). The same QC levels were used for the calculation of the absolute matrix effect, which was determined by analysis of the mean area response of blank matrix spiked with analytes after SPE compared to the mean analyte response of a neat solution using the same analyte concentrations. Furthermore, the CV of the internal standard normalised matrix factor was assessed using blank plasma samples from six healthy volunteers. The matrix factor was calculated for each source after comparing the mean area ratio of blank samples spiked with analytes after SPE to the mean area ratio of a neat solution of analytes at two QC levels (high [750.0 pg/mL] and low [31.1 pg/mL]). The CV was determined for all calculated internal standard normalised matrix factors and was restricted to ≤15% to ensure that the internal standard corrects for individual-dependent matrix effects.

Furthermore, dilution integrity was evaluated for a 1:10 dilution of a 6 ng/mL spiked plasma sample in a fivefold approach. A maximum deviation of 15% (CV and RE) was allowed. Additionally, parallelism was assessed by four serial dilutions of endogenous samples within the calibration range of the assay. As no samples of diseased patients were available, blood was sampled from three healthy volunteers without the addition of the protease inhibitor to allow for the artificial generation of bradykinin and its metabolites. The protease inhibitor was added after 30 min to plasma. Kallidin and des-Arg(10)-kallidin were spiked at a mid-concentration within the calibration range, as these peptides are not artificially generated after blood sampling. Stock samples and dilutions were analysed in triplicate and back-calculated values of the diluted values were compared to undiluted samples, whereby the CV had to be ≤15%.

Finally, stability was examined using three replicates of four QC levels (high [750.0 pg/mL], mid [125.0 pg/mL] and low [31.1 pg/mL and 7.8 pg/mL]). To assess benchtop stability, plasma samples were placed at room temperature for 1.5 h and 3 h before further processing and analysis. Freeze-thaw stability was determined after one, two and four cycles of complete thawing and refreezing of samples at −80 °C. Furthermore, long-term stability of the plasma samples was analysed after 1, 2 and 4 weeks. For all stability investigations, the maximum deviation of the calculated concentration to the nominal concentration compared to a freshly prepared calibration curve was ≤15% (RE).

### Applicability

Endogenous plasma levels of the kinin peptides were determined in three healthy volunteers (2 men, 1 woman). Lower kinin concentrations are expected in healthy volunteers than in patients with kinin-mediated diseases (e.g. angioedema), thus making this investigation serve as an especially stringent measure of the ability of the platform to detect low kinin levels. Blood was sampled under aspiration into EDTA-containing S-Monovettes® prespiked with and without the protease inhibitor. Blood sampling was conducted in the sitting position between 10:30 a.m. and 12:30 p.m. Repeated blood sampling on two distinct days was conducted in two volunteers. Additionally, plasma of healthy volunteers was sampled into BD™ P100 and P800 blood collection tubes (Becton Dickinson, Heidelberg, Germany) to facilitate comparison to previously published kinin levels. Subsequent to blood sampling, samples were centrifuged at 2000×*g* for 10 min at room temperature and plasma was transferred into low protein binding tubes before further processing.

## Results

### Method development

#### Blank plasma generation

**Fig. 3:**
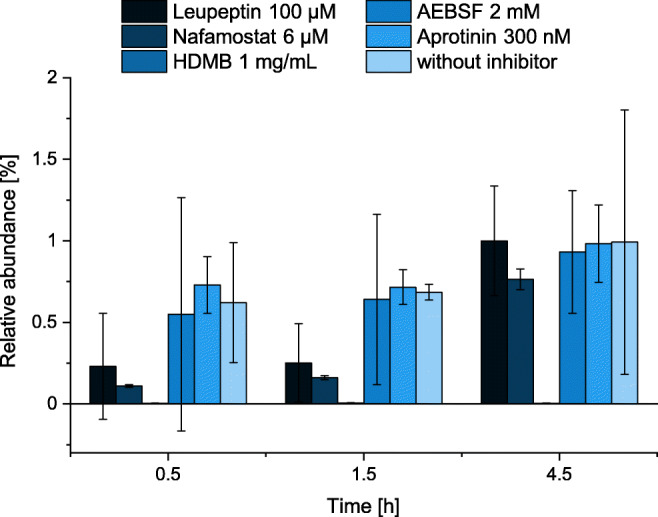
Evaluation of inhibitors preventing the artificial formation of bradykinin for generation of blank plasma. All experiments were conducted in triplicate (mean ± standard deviation). AEBSF, 4-(2-aminoethyl)benzolsulfonylfluoride; HDMB, hexadimethrine bromide

AEBSF and aprotinin alone did not sufficiently prevent the artificial generation of bradykinin (Fig. 3). Additionally, a high CV was observed for AEBSF, caused by one replicate exceeding the other bradykinin values by factor 10, indicating variable inhibition despite the same storage and handling conditions of the replicates. Even after the exclusion of outliers, AEBSF performed inferior to nafamostat and hexadimethrine bromide. While nafamostat and leupeptin only decreased the formation of bradykinin at the 0.5 h and 1.5 h timepoints, the lowest levels of bradykinin were detected with the use of hexadimethrine bromide. Nafamostat and hexadimethrine bromide seemed to be the most promising regarding reproducibility of a blank matrix, as lowest bradykinin levels and CV were observed. Both were applied in combination and their combined use was found to result in blank plasma after 4.5 h (Fig. 2). Further storage at room temperature for 24 h confirmed that the plasma remained blank using this inhibitor combination. The consistency of the results was evaluated in six sources, and blank plasma was successfully generated for each source.

#### Determination of the calibration curve range

By increasing the plasma sample volume loaded on the SPE cavity from 100 to 150 μL, an improvement by the same factor was observed for the LLOQ. Further increasing the sample volume to 300 μL resulted in an equal intensity of bradykinin 1-5, which may have been affected by ion suppression due to increased matrix presence. A calibration curve applying 150 μL of plasma resulted in linear ranges between an LLOQ of 1.3–5.0 pg/mL (depending on the kinin) and an ULOQ of 3000 pg/mL (for all kinins). Repetition of this range and evaluation of precision and accuracy revealed that power or Wagner regression was necessary to include all calibrator levels in a valid run. In addition to matrix effects, non-linearity can result from SPE column overloading and detector saturation due to co-eluting matrix compounds. Hence, to enable more common regression methods, the calibration curve range was reduced to 10 calibrator levels ranging from 2.0 to 1000 pg/mL.

### Validation

#### Linearity

All analytes showed good linearities (mean *r* ≥ 0.998, *n* = 3) through the studied concentration range. Best fits were achieved using 1/*x*^2^ weighting and quadratic regression. The four analytes bradykinin, des-Arg(10)-kallidin, des-Arg(9)-bradykinin and bradykinin 1–7 were linear within a range of 2.0 to 1000.0 pg/mL (10-point calibration line). For the other analytes, the same ULOQs were used, but higher LLOQs were set as follows: bradykinin 1-5: 15.6 pg/mL (7 points), bradykinin 2-9: 7.8 pg/mL (8 points) and kallidin: 3.9 pg/mL (9 points). In molar units, these LLOQs were 1.9 fmol/mL for bradykinin, 2.2 fmol/mL for des-Arg(9)-bradykinin, 2.6 fmol/mL for bradykinin 1-7, 27.2 fmol/mL for bradykinin 1-5, 3.4 fmol/mL for kallidin, 8.6 fmol/mL for bradykinin 2-9 and 1.9 fmol/mL for des-Arg(10)-kallidin. Example chromatograms of the LLOQ and ULOQ are presented in Fig. 2.

#### Accuracy, precision and sensitivity

All RE values were ≤ ±14.6% at all QC levels except for the LLOQ, at which the values were ≤ ±17.1%, thus fulfilling the guideline criteria of the FDA of ≤ ±15% (RE) at the QC levels and ≤ ±20% (RE) at the LLOQ, respectively [25]. Between-run accuracy at the QC high (750.0 pg/mL) and QC mid (125.0 pg/mL) levels was between −3.5 and 1.8% (RE). Between-run variation, used to measure precision, was below 6.9% (CV) at these levels. All precision results for the QC low (7.5–14.7% (CV)) and the LLOQ (10.9–19.8% (CV)) similarly complied with the regulatory guideline of the FDA (≤15% at the QC levels and ≤ 20% at the LLOQ) [25]. Detailed results for within- and between-run accuracy as well as precision are displayed in Table 2. The signal-to-noise ratios at the respective LLOQs were 89:1 for bradykinin 1-5, 144:1 for bradykinin 1-7, 134:1 for kallidin, 214:1 for bradykinin, 93:1 for des-Arg(10)-kallidin, 67:1 for bradykinin 2-9 and 333:1 for des-Arg(9)-bradykinin.

**Table 2:** Accuracy and precision results

Analyte	Nominal concentration (pg/mL)		Accuracy				Precision	
			Day 1 RE (%)	Day 2 RE (%)	Day 3 RE (%)	Between-run RE (%)	Within-run CV (%)	Between-run CV (%)
Kallidin	QC high	750.0	1.1	−1.5	−2.1	−0.8	3.1	3.2
	QC mid	125.0	−0.9	−4.6	−1.6	−2.4	3.3	3.6
	QC low	7.8	−8.0	−8.5	−11.1	−9.2	8.9	8.9
	LLOQ	3.9	−14.6	−9.0	−12.7	−12.1	10.9	10.9
Bradykinin	QC high	750.0	1.9	−1.5	−1.9	−0.5	3.4	3.7
	QC mid	125.0	−7.0	−0.2	−3.1	−3.4	2.0	3.9
	QC low	3.9	13.2	2.4	−5.4	3.4	10.5	13.0
	LLOQ	2.0	8.0	−4.4	−9.0	−1.8	15.2	16.3
Des-Arg(10)-kallidin	QC high	750.0	2.2	1.9	1.4	1.8	3.0	3.0
	QC mid	125.0	−5.7	0.8	2.8	−0.7	2.7	5.1
	QC low	3.9	2.7	−11.6	9.7	0.3	11.2	14.7
	LLOQ	2.0	5.4	−5.7	3.8	1.2	18.4	18.4
Des-Arg(9)-bradykinin	QC high	750.0	2.7	−4.5	−0.5	−0.8	2.5	4.3
	QC mid	125.0	3.2	−5.6	5.5	1.1	2.4	6.2
	QC low	3.9	1.6	14.5	−5.9	3.4	7.7	12.1
	LLOQ	2.0	−1.6	11.9	−17.1	−2.3	14.7	19.8
Bradykinin 2-9	QC high	750.0	4.7	−0.3	−0.2	1.4	2.3	3.5
	QC mid	125.0	2.4	−4.3	6.5	1.5	4.8	6.9
	QC low	15.6	13.8	−0.2	9.9	7.8	9.8	11.0
	LLOQ	7.8	13.5	−3.9	−4.2	1.8	15.4	17.0
Bradykinin 1-7	QC high	750.0	1.1	1.9	−1.9	0.4	2.3	2.8
	QC mid	125.0	−1.6	−1.6	0.2	−1.0	3.0	3.0
	QC low	3.9	−4.5	−2.7	−6.5	−4.6	7.5	7.5
	LLOQ	1.9	−4.5	−3.5	−16.9	−8.3	13.6	14.6
Bradykinin 1-5	QC high	750.0	−2.9	−1.0	−5.2	−3.0	2.7	3.2
	QC mid	125.0	0.1	−4.4	−0.4	−1.6	5.3	5.4
	QC low	31.1	9.5	−5.7	−4.6	−0.2	12.3	13.9
	LLOQ	15.6	−6.8	1.9	−8.6	−4.5	11.9	12.2

*CV* coefficient of variation, *LLOQ* lower limit of quantification, *QC* quality control, *RE* relative error

#### Carry-over

The carry-over in a blank following an ULOQ sample was below 20% for all analytes: 0.0% for bradykinin 1-5, 5.4% for kallidin, 3.2% for bradykinin 1-7, 5.4% for bradykinin, 1.7% for des-Arg(10)-kallidin, 5.1% for bradykinin 2-9 and 10.8% for des-Arg(9)-bradykinin. For the internal standard, no carry-over was observed.

#### Recovery and matrix effects

The mean recovery at all distinct QC levels investigated (*n* = 4 levels, *n* = 3 replicates) was above 90% for the four rather lipophilic peptides bradykinin (91.1%), des-Arg(10)-kallidin (95.5%), bradykinin 2-9 (92.0%) and des-Arg(9)-bradykinin (97.2%). Mean recovery was 69.5% for bradykinin 1-5, 88.2% for kallidin and 78.7% for bradykinin 1-7 (Table 3). Lower recoveries were observed for the more hydrophilic peptides (bradykinin 1-5, bradykinin 1-7, kallidin) with fewer basic functional groups (bradykinin 1-7, bradykinin 1-5), presumably because of less reversed phase retention and ion exchange with the acidic carboxyl groups of the WCX SPE material. The mean absolute matrix effect for all distinct QC levels (*n* = 4 levels, *n* = 3 replicates) was −54.0% for bradykinin 1-5, −26.6% for kallidin, −27.3% for bradykinin 1-7, −13.8% for bradykinin, −17.3% for des-Arg(10)-kallidin, −27.3% for bradykinin 2-9 and − 16.1% for des-Arg(9)-bradykinin (Table 3).

**Table 3:** Absolute matrix effect, CV of internal standard normalised matrix factor of 6 human sources and recovery for all kinin peptides

**Analyte**	**Absolute matrix effect**			
	*QC high* *(750.0 pg/mL)*	*QC mid* *(125.0 pg/mL)*	*QC low* *(31.1 pg/mL)*	*QC low* *(7.8 pg/mL)*
**Bradykinin 1-5**	−54.3%	−54.9%	−52.9%	-
**Kallidin**	−25.5%	−27.6%	−27.5%	−25.6%
**Bradykinin 1-7**	−27.1%	−30.7%	−29.5%	−22.0%
**Bradykinin**	−19.0%	−11.4%	−13.4%	−11.2%
**Des-Arg(10)-kallidin**	−20.7%	−16.9%	−14.2%	−17.3%
**Bradykinin 2-9**	−31.3%	−24.8%	−25.7%	-
**Des-Arg(9)-bradykinin**	−26.3%	−28.0%	−27.3%	−16.1%
**Analyte**	**Internal standard normalised matrix factor (CV,** ***n*** **= 6)**			
	*QC high* *(750.0 pg/mL)*		*QC low* *(31.1 pg/mL)*	
**Bradykinin 1-5**	11.2%		10.3%	
**Kallidin**	1.9%		3.6%	
**Bradykinin 1-7**	0.8%		3.5%	
**Bradykinin**	2.1%		4.8%	
**Des-Arg(10)-kallidin**	2.0%		2.5%	
**Bradykinin 2-9**	1.5%		6.6%	
**Des-Arg(9)-bradykinin**	1.0%		2.7%	
**Analyte**	**Recovery**			
	*QC high* *(750.0 pg/mL)*	*QC mid* *(125.0 pg/mL)*	*QC low* *(31.1 pg/mL)*	*QC low* *(7.8 pg/mL)*
**Bradykinin 1-5**	73.3%	60.9%	74.3%	-
**Kallidin**	90.8%	87.0%	79.4%	95.7%
**Bradykinin 1-7**	80.2%	74.6%	82.0%	78.0%
**Bradykinin**	93.2%	87.9%	91.0%	92.1%
**Des-Arg(10)-kallidin**	93.8%	91.7%	91.4%	105.2%
**Bradykinin 2-9**	95.5%	88.3%	92.4%	-
**Des-Arg(9)-bradykinin**	97.7%	98.8%	93.9%	98.5%

*CV* coefficient of variation, *QC* quality control

The CV of the internal standard normalised matrix factor was below 15% at the investigated QC levels using plasma from six volunteers and thus confirmed no inter-source variability (Table 3). At the QC high level (750.0 pg/mL), the CV of the internal standard normalised matrix factors was 2.1% for bradykinin, 1.9% for kallidin, 1.0% for des-Arg(9)-bradykinin, 2.0% for des-Arg(10)-kallidin, 0.8% for bradykinin 1-7, 1.5% for bradykinin 2-9 and 11.2% for bradykinin 1-5. At the QC low level (31.1 pg/mL), the CV was 4.8% for bradykinin, 3.6% for kallidin, 2.7% for des-Arg(9)-bradykinin, 2.5% for des-Arg(10)-kallidin, 3.5% for bradykinin 1-7, 6.6% for bradykinin 2-9 and 10.3% for bradykinin 1-5. Haemolysed and lipaemic samples were not assessed as part of specificity investigations.

#### Dilution integrity and parallelism

Dilution integrity was confirmed for a 1:10 dilution of a 6 ng/mL plasma sample. Accuracy (RE) and precision (CV) were as follows for the kinin peptides: −13.0% (RE)/4.9% (CV) for bradykinin 1-5, −2.2%/0.2% for kallidin, −5.5%/2.5% for bradykinin 1-7, −1.7%/2.5% for bradykinin, −2.0%/2.0% for des-Arg(10)-kallidin, −10.1%/3.2% for bradykinin 2-9 and − 4.7%/2.7% for des-Arg(9)-bradykinin.

Endogenously generated plasma levels for the assessment of parallelism varied interindividually for each kinin throughout the whole calibration curve range. The CV of the back-calculated concentrations of the dilutions was between 1.7 and 12.8% for all kinins and the three sources and therefore guideline-compliant. Depending on the observed low concentrations in undiluted samples, levels could not be determined for all dilution steps. Details are provided in Table 4.

**Table 4:** Parallelism of the kinin peptides. Values of endogenous/spiked plasma samples as stock concentration and back-calculated values of diluted samples

Analyte		Source 1	Source 2	Source 3
Kallidin	Undiluted concentration	161.9 pg/mL	139.1 pg/mL	155.6 pg/mL
	Dilution factor	Back-calculated concentration (pg/mL)		
	2	162.2	129.5	164.9
	4	157.0	134.2	163.5
	8	157.5	157.7	171.1
	16	137.9	174.9	151.9
	CV (%)	6.5	12.8	3.9
Bradykinin	Undiluted concentration	733.7 pg/mL	63.9 pg/mL	294.7 pg/mL
	Dilution factor	Back-calculated concentration (pg/mL)		
	2	712.9	59.7	305.5
	4	697.9	72.2	315.3
	8	691.0	75.0	325.8
	16	700.9	85.9	323.8
	CV (%)	2.4	14.3	4.0
Des-Arg(10)-kallidin	Undiluted concentration	230.4 pg/mL	214.9 pg/mL	208.4 pg/mL
	Dilution factor	Back-calculated concentration (pg/mL)		
	2	229.1	199.6	229.6
	4	228.1	201.8	212.1
	8	225.8	238.7	222.9
	16	220.5	204.3	236.6
	CV (%)	1.7	7.6	6.1
Des-Arg(9)-bradykinin	Undiluted concentration	812.2 pg/mL	39.5 pg/mL	138.2 pg/mL
	Dilution factor	Back-calculated concentration (pg/mL)		
	2	852.1	39.5	137.6
	4	829.7	44.5	138.3
	8	849.9	47.8	130.6
	16	892.5	< LLOQ	118.7
	CV (%)	3.5	9.5	7.3
Bradykinin2-9	Undiluted concentration	114.9 pg/mL	9.4 pg/mL	66.7 pg/mL
	Dilution factor	Back-calculated concentration (pg/mL)		
	2	103.4	< LLOQ	58.0
	4	130.4	< LLOQ	71.2
	8	119.1	< LLOQ	53.3
	16	104.8	< LLOQ	< LLOQ
	CV (%)	9.7	n/a	10.8
Bradykinin 1-7	Undiluted concentration	196.9 pg/mL	7.3 pg/mL	69.6 pg/mL
	Dilution factor	Back-calculated concentration (pg/mL)		
	2	190.0	3.8	72.2
	4	185.3	2.0	67.6
	8	177.8	< LLOQ	69.9
	16	176.1	< LLOQ	74.8
	CV (%)	4.6	4.8	4.4
Bradykinin 1-5	Undiluted concentration	28.8 pg/mL	< LLOQ	25.4 pg/mL
	Dilution factor	Back-calculated concentration (pg/mL)		
	2	34.1	< LLOQ	< LLOQ
	4	< LLOQ	< LLOQ	< LLOQ
	8	< LLOQ	< LLOQ	< LLOQ
	16	< LLOQ	< LLOQ	< LLOQ
	CV (%)	12.0	n/a	n/a

*CV* coefficient of variation, *LLOQ* lower limit of quantification, *n/a* not applicable

#### Stability

All analytes were stable for 1.5 h on the benchtop. After 3 h, low concentrations of bradykinin and bradykinin 1-7 and high and mid concentrations of des-Arg(9)-bradykinin were outside the set limit of ≤ ±15%. Freeze-thaw stability was confirmed for one and two freeze-thaw cycles for every kinin peptide. In contrast to the other analytes, which were also stable for four freeze-thaw cycles, bradykinin 1-7 and bradykinin 1-5 trended towards a decrease. Long-term stability at −80 °C was confirmed for a period of four weeks for all peptides (maximum period of investigation). Detailed results are provided in Table 5.

**Table 5:** Results of the stability assessments

Analyte	Nominal concentration (pg/mL)		Benchtop stability		Freeze-thaw stability			Long-term stability
			1.5 h at 21 °C RE (%)	3 h at 21 °C RE (%)	1 cycle RE (%)	2 cycles RE (%)	4 cycles RE (%)	4 weeks RE (%)
Kallidin	QC high	750.0	−3.3	−9.6	1.1	−7.2	−10.3	−6.7
	QC mid	125.0	−1.7	−10.8	−9.5	−2.1	−2.7	8.6
	QC low	31.1	−4.4	−0.7*	−4.4	1.4	−5.3	6.6
	QC low	7.8	0.0	−0.8	12.4	−14.9	−6.5	14.9
Bradykinin	QC high	750.0	−1.4	−7.4	0.4	−7.8	−8.3	−3.0
	QC mid	125.0	−1.2	1.2	−3.2	−6.2	−10.8	5.9
	QC low	31.1	1.4	8.4*	−1.5	−6.2	−9.8	10.0
	QC low	7.8	4.9	138.0	−1.5	1.6	−7.3	4.5
Des-Arg(10)-kallidin	QC high	750.0	−0.9	−11.2	6.1	−8.4	−7.9	−2.1
	QC mid	125.0	6.9	−2.4	−0.8	−7.7	−7.7	0.9
	QC low	31.1	13.1	−7.9*	−1.2	0.4	−8.6	13.8
	QC low	7.8	9.7	9.4	−11.8	0.8	3.5	10.6
Des-Arg(9)-bradykinin	QC high	750.0	−4.6	−21.8	−0.3	−8.4	−7.9	0.5
	QC mid	125.0	1.1	−16.1	2.1	−7.7	−7.7	3.3
	QC low	31.1	3.4	−5.2*	5.4	0.4	−8.6	12.3
	QC low	7.8	−10.2	11.8	−11.7	0.8	3.5	8.2
Bradykinin 2-9	QC high	750.0	−3.3	−9.1	−1.1	−7.2	−10.6	−0.6
	QC mid	125.0	−2.4	−8.0	−0.5	−12.2	−11.7	1.7
	QC low	31.1	4.7	3.8*	11.8	−4.7	−0.5	9.0
Bradykinin 1-7	QC high	750.0	1.7	2.1	2.0	−6.6	−7.0	−2.6
	QC mid	125.0	9.0	3.6	−1.3	−2.6	−6.1	6.0
	QC low	31.1	3.1	10.7*	4.0	−6.3	−15.3	7.5
	QC low	7.8	11.8	16.0	−7.3	0.3	−12.3	13.6
Bradykinin 1-5	QC high	750.0	−0.6	−7.6	4.0	−8.0	−20.3	−11.0
	QC mid	125.0	−1.1	−2.6	−8.3	−5.8	−14.1	11.3
	QC low	31.1	−8.7	−5.3*	−5.4	−2.8	−30.6	13.9

*n = 1; *QC* quality control, *RE* relative error

### Applicability

**Fig. 4:**
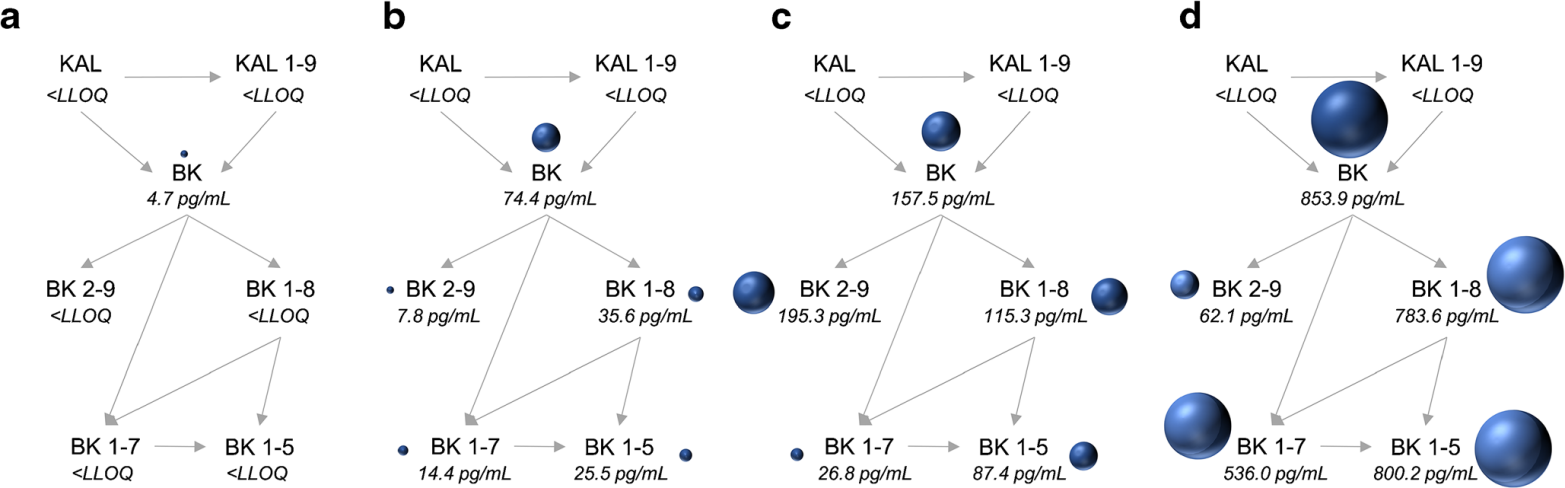
Comparison of distinct protease inhibitor approaches. Kinin levels were determined in prespiked Monovettes® (**a**), in BDTM P100 tubes (**b**) and in BDTM P800 tubes (**c**), and without the use of inhibitors (**d**). Measured endogenous median levels of kinin peptides (n=3) are displayed. The bullet size reflects the amount of kinin peptides measured. BK, bradykinin; KAL, kallidin

Endogenous plasma levels of three healthy volunteers ranged between 2.3 and 6.8 pg/mL (median 4.7 pg/mL) for bradykinin. Des-Arg(9)-bradykinin was detected in one male source at a concentration of 3.2 pg/mL, whereas the other sources showed levels below the LLOQ (2 pg/mL). Similarly, des-Arg(10)-kallidin was quantified in one female source, at a concentration of 3.2 pg/mL. Bradykinin 1-7, bradykinin 2-9, bradykinin 1-5 and kallidin were below the quantification limit. An example chromatogram is shown in Fig. 2. The reproducibility of measured kinin levels was confirmed on two distinct days in two human sources (median 5.8 pg/mL bradykinin). The ready-to-use BD™ P100 and P800 tubes also reduced the artificial generation/degradation of bradykinin. However, the inhibitory power was less than the one of prespiked S-Monovettes® and subsequently, the generation was less controlled being reflected by higher levels of detected bradykinin levels (median 74.4 pg/mL using P100 and 157.5 pg/mL using P800) (Fig. 4). In contrast, substantially higher levels of bradykinin and its metabolites were observed using collection devices without any inhibitor (Fig. 4). Increases of determined levels of factor 62 up to 800 were observed. The experiments using non-inhibited samples were conducted as fast as possible at room temperature, whereby the time period from blood collection, transport, 10-min centrifugation until loading of plasma on the SPE at the bench took about 20 min.

## Discussion

A sensitive and targeted LC-MS/MS platform for the comprehensive determination of the active kinins with their major metabolites was successfully validated according to the regulatory bioanalytical guideline of the FDA [25]. A broad calibration curve range covering expected concentrations of kinins in disease, with LLOQs in the low pg/mL range, was established. No source-dependent matrix effects were identified, and suitable stability of the analytes in plasma was observed.

The validated kinin peptide platform represents a novel tool that enables the determination of kinins in plasma with clearly improved sensitivity and with a comprehensiveness that has not been previously published. While low detection limits (0.4–11.7 pg/mL) have been claimed for immunoassays [2, 15, 27], these lack specificity due to cross-reactions owing to structural similarities (Fig. 1). This leads to the requirement for extensive sample clean-up, including chromatographic separation and multiple SPEs, prior to quantification by immunoassay [2, 28]. However, to date, reported quantification limits for bradykinin by LC-MS/MS are quite high—namely, between 94 pg/mL [19] up to 10 ng/mL in plasma or serum [20, 21, 29]. The presented assay shows a far more sensitive quantification limit of 2 pg/mL for bradykinin. Preceding investigations using a design of experiments approach identified non-specific peptide adsorption and the usefulness of modifiers (3.2% DMSO and 0.1% formic acid) in the mobile phase as highly impacting factors concerning the assay’s sensitivity [30]. Despite the distinct physicochemical properties of the peptides, an adjustment within the design space allowed for the identification of an injection solvent (10/10/80 formic acid/methanol/water [v/v/v]) reducing non-specific adsorption for all kinins [31]. For example, quantification limits of 2 ng/mL were achieved for des-Arg(9)-bradykinin by van den Broek et al. in 2010 [21]; thus, the presented assay marks an improvement by a factor of 1000. Furthermore, current LC-MS/MS assays or immunoassays require at least 500 μL of plasma/1 mL of blood to establish these (low) quantification limits, whereas the presented assay requires only 150 μL of plasma [2, 19, 21, 28, 29]. This reduction enables repeated blood sampling in severely ill patients without increasing the risk of anaemia. Additionally, to the best of our knowledge, validated LC-MS/MS assays are not yet on hand for bradykinin 1-7, bradykinin 2-9, kallidin and des-Arg(10)-kallidin in plasma. Thus, with this study, the sensitive, comprehensive and simultaneous determination of all active kinin peptides with their major metabolites is facilitated for the first time in plasma.

An ideal surrogate matrix should closely resemble the study samples and be analyte-free [32]. Previous LC-MS/MS assays for bradykinin used water [19] or bovine plasma as a surrogate matrix due to the endogenous presence of kinins in human matrix [21]. However, different compositions of a surrogate matrix in comparison to study samples might lead to matrix effects affecting the accuracy of the results [32]. Therefore, a better approach is provided by the use of standard addition for bradykinin, as reported by Lame et al. 2016 [20]. However, the principle of standard addition becomes labour-intensive if multiple samples are to be analysed, and it requires a large volume of the patient sample due to the preparation of calibration curves for each study sample. A special feature of the established platform is the use of human blank plasma for calibration curves and QC samples. The use of blood from a volunteer with endogenous levels of bradykinin 1-5 below the detection limit as blank matrix [22] resembles the approach used within this study. However, in this study, blank plasma was actively generated and depletion of kinins by the applied procedure was successfully confirmed in six volunteers. This enables cost-effective blank generation regardless of the availability of a particular source.

All analytes were stable for 1.5 h on the benchtop, for 4 weeks at −80 °C (indicating long-term stability) and for at least two freeze-thaw cycles at the distinct QC levels. This confirms the suitability of the preanalytical conditions and the prevention of artificial generation or degradation of peptides throughout distinct concentration ranges. Stability experiments for kinin peptides in plasma are rare. Lindström et al. reported long-term stability of a 106 ng/mL sample for 1 year, but freeze-thaw stability was not observed, as indicated by a 19% decrease of the nominal concentration after one freeze-thaw cycle and a 45% decrease after a second cycle (FDA limit: ≤ ± 15%) [19, 25]. van den Broek et al. (2010) found bradykinin (114 ng/mL) and des-Arg(9)-bradykinin (8 ng/mL) to be stable for 1 h on ice, for 5 months at −80 °C and for three freeze-thaw cycles [21]. Lower concentrations, as assessed in this study, have not yet been investigated. However, at these concentrations, instability due to insufficient inhibition of degrading enzymes becomes obvious. For example, generation of bradykinin has been observed after 3 h on the benchtop at the lowest QC level, whereas at higher concentrations, it was not identifiable. Insufficient suppression of artificial generation due to instability of the protease inhibitor at room temperature may be causative. These results reveal that stability investigations covering the entire calibration curve range are essential.

The endogenous kinin concentrations measured (in the low pg/mL range) were in the range of levels determined by immunometric detection. Campbell et al. (1993) reported levels of 2.0 pg/mL for bradykinin, <0.8 pg/mL for des-Arg(9)-bradykinin (< LLOQ), and < 1 pg/mL for bradykinin 1-7 (< LLOQ) in healthy volunteers (*n* = 12) [28]. Duncan et al. (2000) reported levels of <0.4 pg/mL for bradykinin (< LLOQ), 0.6 pg/mL for des-Arg(9)-bradykinin, 1.8 pg/mL for bradykinin 1-7, and < 0.5 pg/mL for kallidin and des-Arg(10)-kallidin (< LLOQ) (*n* = 8–11) [2]. In contrast, much higher values were determined by Lindström et al., who reported plasma bradykinin levels between 530 and 1166 pg/mL by LC-MS/MS [19]. In addition, van den Broek et al. reported concentrations of 57–162 ng/mL of bradykinin and 50–151 ng/mL of des-Arg(9)-bradykinin in serum from healthy controls in [21]. Lame et al. (2013 and 2016) compared distinct commercially available inhibitors and found reduced plasma levels of 90.0 pg/mL [33] and 186.0 pg/mL [20] of bradykinin using BD™ P100 tubes as compared to the use of no inhibitors (810.7 pg/mL [20]). Levels in BD™ P800 plasma could not be determined owing to degradation of the internal standard (des-Arg(10)-kallidin) in these samples [20]. Similarly, in this study, median levels of 74.4 pg/mL of bradykinin using BD™ P100 tubes were observed, which were substantially lower compared to the use of no inhibitor, where levels of 853.9 pg/mL were determined. However, elevated levels of bradykinin and its metabolites in comparison to the prespiked S-Monovettes® indicate incomplete inhibition of kinin metabolism by the protease inhibitor in BD™ P100/P800 tubes. Insufficient prevention of factor XII–mediated artificial bradykinin generation might be causative for the observed varying kinin levels, especially if no protease inhibitor was added. This makes careful stability assessments, as discussed above, crucial for reliable quantification results.

While concentrations of kinins are low in healthy individuals, in patients with kinin-mediated diseases, they are expected to be increased; thus, the established platform is capable of quantifying clinically relevant concentrations. The developed platform is especially useful for extending the knowledge about diseases in which the KKS in plasma plays a substantial role in their pathophysiology. This includes, for example, sepsis and angioedema, in which activation of the KKS is assumed to be causative for vasodilation, inflammation and oedema formation [34, 35]. Despite strong evidence in animal models for a link between coagulopathy, the KKS and septic shock, evidence in humans is limited and needs further investigation [34]. In severe COVID-19, elevated D-dimers, in conjunction with a strong decrease in platelet count, indicate an increased activation of the coagulation system [36, 37]. Due to the strong correlation of the KKS to the intrinsic coagulation system via the activation of plasma kallikrein by factor XII, subsequently elevated active kinin levels might explain symptoms like inflammation, cough, diarrhoea, anosmia and capillary leakage [9, 38]. Additionally, alternative bradykinin-forming pathways might develop, and des-Arg(9)-bradykinin degradation by ACE 2 might be impaired [9, 39]. RNA sequence analysis has shown that plasma kallikrein and bradykinin precursors are expressed at increased levels and that degrading enzymes (e.g. ACE) are substantially downregulated in patients with COVID-19 [5]. The simultaneous analysis of bradykinin and kallidin in conjunction with their metabolites in plasma is facilitated by the developed LC-MS/MS platform, making it possible to shed light on the consequences of the detected alterations at the RNA level on the active kinin peptides, as well as their potentially altered degradation. If exploratory investigations confirm the usefulness of kinins as biomarkers, further steps towards a full validation will be undertaken using patient samples (e.g. extensive investigation of matrix effects, selectivity, parallelism, stability, relative accuracy) [40, 41].

## Conclusion

A sensitive and targeted LC-MS/MS platform was established and validated according to the bioanalytical guideline of the FDA for bradykinin 1-5, bradykinin 1-7, bradykinin 2-9, des-Arg(9)-bradykinin, kallidin, bradykinin and des-Arg(10)-kallidin. Quantification limits in the low pg/mL range in conjunction with a broad calibration curve range were established. The platform was successfully applied to determine endogenous levels of kinin peptides in plasma of healthy volunteers. It facilitates the simultaneous determination of the major kinins and allows the generation of a more complete picture of the KKS in diseases in which it plays a role, such as angioedema and COVID-19.
